# Characterization of hypothetical proteins Cpn0146, 0147, 0284 & 0285 that are predicted to be in the *Chlamydia pneumoniae *inclusion membrane

**DOI:** 10.1186/1471-2180-7-38

**Published:** 2007-05-15

**Authors:** Jianhua Luo, Guangchao Liu, Youmin Zhong, Tianjun Jia, Kaiyang Liu, Ding Chen, Guangming Zhong

**Affiliations:** 1 Department of Microbiology and Immunology, University of Texas Health Science Center at San Antonio, 7703 Floyd Curl Drive, San Antonio, TX 78229, USA; 2 Life Science Research Center, Hebei North University, 14 Changqing Road, Zhangjiakou, Hebei 075029, PR of China

## Abstract

**Background:**

Although more than 100 *Chlamydia pneumoniae *hypothetical proteins have been predicted to be inclusion membrane proteins, only a few have been experimentally demonstrated to be in the inclusion membrane. Using antibodies raised with fusion proteins, we characterized four such hypothetical proteins encoded by two gene clusters (*Cpn0146-147 *and *Cpn0284-285*) in the *C. pneumoniae *genome.

**Results:**

Cpn0146 and 0147 were detected in the inclusion membrane while Cpn0284 and 0285 inside inclusion and mainly associated with reticulate bodies although all four proteins contain an N-terminal bi-lobed hydrophobic region, a signature motif assigned to inclusion membrane proteins. These four hypothetical proteins were only detected in cells infected with *C. pneumoniae *but not other chlamydial species, with Cpn0147 at 6 hours and Cpn0146, 0284 & 0285 at 24 hours after infection. Cpn0146 & 147 but not Cpn0284 and 285 co-localized with a host cell endoplasmic reticulum marker, a property known to be possessed by some chlamydial inclusion membrane proteins, when expressed in the host cell cytosol via transgenes. However, the endoplasmic reticulum localization of the *C. pneumoniae *inclusion membrane proteins did not result in inhibition of the subsequent *C. pneumoniae *infection.

**Conclusion:**

The hypothetical proteins Cpn0146 & 0147 were localized in the *C. pneumoniae *inclusion membrane while Cpn0284 & 0285 within the inclusion although all four were predicted to be Inc proteins, suggesting the need to experimentally characterize the predicted Inc proteins.

## Background

The obligate intracellular chlamydial pathogens include the species *Chlamydia trachomatis *(*C. trachomatis*; [1]) and *C. pneumoniae *[2] that mainly infect humans and *C. muridarum *(formerly known as *C. trachomatis *mouse pneumonitis agent, designated as MoPn, ref: [2]), *C. caviae *[3], *C. psittaci *(38), *C. abortus *[4] and *C. felis *[5] that are mainly animal pathogens. The species *C. pneumoniae*, *C. caviae*, *C. psittaci*, *C. abortus *&*C. felis *are also grouped as an independent genus termed Chlamydophilae based on their genetic relatedness [6]. The *C. pneumoniae *organisms infect the human respiratory system, not only causing respiratory pathologies but also exacerbating pathologies in other organs such as the vascular wall [7-10]. The *C. caviae *GPIC organisms can infect both the ocular and urogenital tissues in guinea-pig, which has been used as a model system for studying the pathogenesis of Chlamydia-induced diseases [11]. The *C. psittaci *6BC organisms cause avian chlamydiosis that can lead to serious health problems for humans who are in close contact with the infected birds [12]. Both the *C. abortus *&*C. felis *organisms can affect the health of various domesticated animal species [4,13,14]. Despite the profound difference in host range, tissue tropism, disease process, all chlamydial species share similar genome sequences [1-5] and possess a common intracellular growth cycle with distinct biphasic stages [15].

Chlamydial organisms have adapted an obligate intravacuolar growth life style with a two-phase cycle [16,17]. The infection starts with endocytosis of an infectious elementary body (EB) into a host cell, followed by rapid differentiation of the EB into a non-infectious but metabolically active reticulate body (RB). After the RB undergoes numerous rounds of replication, the progeny RBs can differentiate back into EBs before exiting to infect the adjacent cells. Chlamydial organisms accomplish all their biosynthesis and particle assembly within the cytoplasmic vacuole (designated as inclusion). The chlamydial inclusions not only support chlamydial replication but also protect the replicating organisms from host defense mechanisms such as lysosomal fusion [15,18]. At the same time, Chlamydia must import nutrients and metabolic intermediates from host cells into the inclusions [19,20]. However, the molecular mechanisms by which Chlamydia organisms interact with host cells are largely unknown. The fact that Chlamydia-encoded proteins are found in the inclusion membrane (designated as Inc; [21]) suggests that the Inc proteins may participate in the chlamydial interactions with host cells [22,23]. Therefore, searching for and characterization of novel inclusion membrane proteins may provide important information for understanding chlamydial pathogenic mechanisms.

Various approaches have been utilized to identify chlamydial Inc proteins, including direct antibody detection [21,24-27], accessibility to host cell cytoplasm immune proteasome processing [28,29], secretion by heterologous type III secretion systems [30,31] and common structural feature-based computer predictions [32,33]. Although a total of 104 hypothetical proteins encoded in *C. pneumoniae *genome were predicted to be Inc proteins by computer programs [32,33], only a few were proven to be in the inclusion membrane of the *C. pneumoniae*-infected cells by direct antibody labeling [32]. Since not all Inc proteins can be identified by computer prediction and not all predicted Inc proteins are localized in the inclusion membrane of chlamydial organism-infected cells [29,32], it is critical to use experimental approaches to confirm the localization of the putative Incs and to further characterize the Inc proteins. In the current study, we detected the hypothetical proteins Cpn0146 & 0147 in the *C. pneumoniae *inclusion membrane and Cpn0284 & 0285 within the inclusion although all four were predicted to be Inc proteins [32,33]. Furthermore, Cpn0146 & 0147 but not Cpn0284 & 0285 co-localized with a host cell endoplasmic reticulum (ER) marker when expressed via transgenes although the ER co-localization did not significantly affect the subsequent *C. pneumoniae *infection.

## Results

### 1. Localization of Cpn0146 and 0147 in the inclusion membrane and Cpn0284 and Cpn0285 within the inclusion of *C. pneumoniae*-infected cells

**Figure 1:**
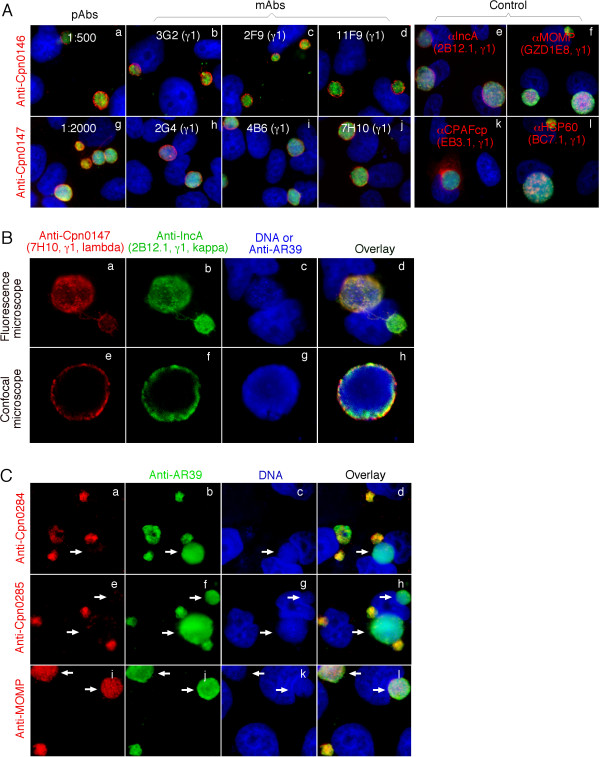
**Localization of Cpn0146, 0147, 0284 & 0285 in *C. pneumoniae*-infected cells**. HeLa cells were infected with *C. pneumoniae *AR39 organisms at an MOI of 0.5 in the presence of 2 μg/ml of cycloheximide for 72 hours. The infected cultures grown on coverslips were processed for various immunostainings. (A) Cpn0146 was probed with a mouse antiserum (pAb, panel a) and monoclonal antibodies (mAb clones 3G2, panel b, 2F9, panel c, 11F9, panel d) while Cpn0147 by pAb (panel g) and mAbs 2G4 (panel h), 4B6 (panel i) and 7H10 (panel j), all of which were raised with the correspodning GST fusion proteins and visualized with a Cy3-conjugated goat anti-mouse IgG (red). Control mouse antibodies against IncA (2B12.1, IgG1; panel e), MOMP (GZD1E8, IgG1, panel f), CPAFcp (EB3.1, IgG1, panel k), and HSP60 (BC7.1, IgG1, panel l) were used to visualize the corresponding antigens. A rabbit anti-AR39 antiserum (R12AR39) together with a Cy2-conjugated goat anti-rabbit IgG (green) was used to visualize the *C. pneumoniae *organisms and Hoechst to visualize DNA. (B) The AR39 organism-infected cell samples were co-stained with the anti-Cpn0147 mAb 7H10 (IgG1, lambda; panel a; red), the anti-incA mAb 2B12.1 (IgG1, kappa; panel b; green) and DNA Hoechst dye (panel c; blue). The triple Images of the immunostainings were obtained using an AX70 fluorescence microscope equipped with a CCD camera. For confocal microscopic observation (panels e to h), the inclusions were visualized with the rabbit antiserum R12AR39 in combination with a goat anti-mouse IgG conjugated with Cy5 (blue, panel g). The images were acquired sequentially one color at a time and overlayed in tri-color using a confocal microscope (Olympus, provided by the UTHSCSA imaging core). Note that the anti-Cpn0146 and 0147 antibodies detected strong inclusion membrane signals similar to and partially overlapped with that obtained with the anti-IncA but not the other reference antibodies. (C) The labelings of Cpn0284 and 0285 with the corresponding pAbs were carried out similarly as described in (A). Note that the anti-Cpn0284 and 0285 antibodies labeled strong signals inside small but not large inclusions (panels a-d for Cpn0284 and e-h for Cpn0285; white arrows pointing to large inclusions) while the anti-MOMP mAb GZD1E8 labeled all inclusions regardless of size (panels i and l).

Using antibodies raised with *C. pneumoniae *fusion proteins, we detected the hypothetical proteins Cpn0146 and 0147 in the inclusion membrane while Cpn0284 and 0285 within the inclusion of *C. pneumoniae*-infected cells (Fig. 1). Both pAb and mAb antibodies against either Cpn0146 or Cpn0147 consistently detected a dominant inclusion membrane signal similar to the signal revealed by the anti-IncA, but not the anti-CPAFcp [34,35], anti-MOMP [36] or anti-HSP60 antibodies (Fig. 1A). We further took advantage of the isotype difference in the light chains between the anti-Cpn0147 mAb 7H10 (lambda) and anti-IncA mAb 2B12.1 (kappa) to co-label these two proteins in the same samples and found that Cpn0147 and IncA partially overlapped with each other under both conventional fluorescence and confocal microscopes (Fig. 1B). Since IncA, encoded by the *C. pneumoniae *ORF *cpn0186*, is a known inclusion membrane protein [32,37], the above observations suggest that Cpn0146 and 0147 are also inclusion membrane proteins. Interestingly, the antibodies raised with Cpn0284 and 0285 fusion proteins labeled dominant signals within the inclusions (Fig. 1C), similar but not identical to the signals revealed by the anti-MOMP or anti-HSP60 antibodies. It is worth noting that the anti-Cpn0284 and 0285 antibodies only detected strong signals in small but not large inclusions (Fig. 1C, panels a-h) while both the anti-MOMP and anti-HSP60 antibodies detected all inclusions regardless of size (Fig. 1A, right panels & Fig. 1C, panels i-l). Since the small inclusions are mainly full of RBs while large inclusions full of EBs under the experimental conditions (72 hours after *C. pneumoniae *infection in the presence of cycloheximide), we can speculate that Cpn0284 and 0285 are likely to be RB-specific proteins.

### 2. Specificity of the anti-chlamydial fusion protein antibodies

**Figure 2:**
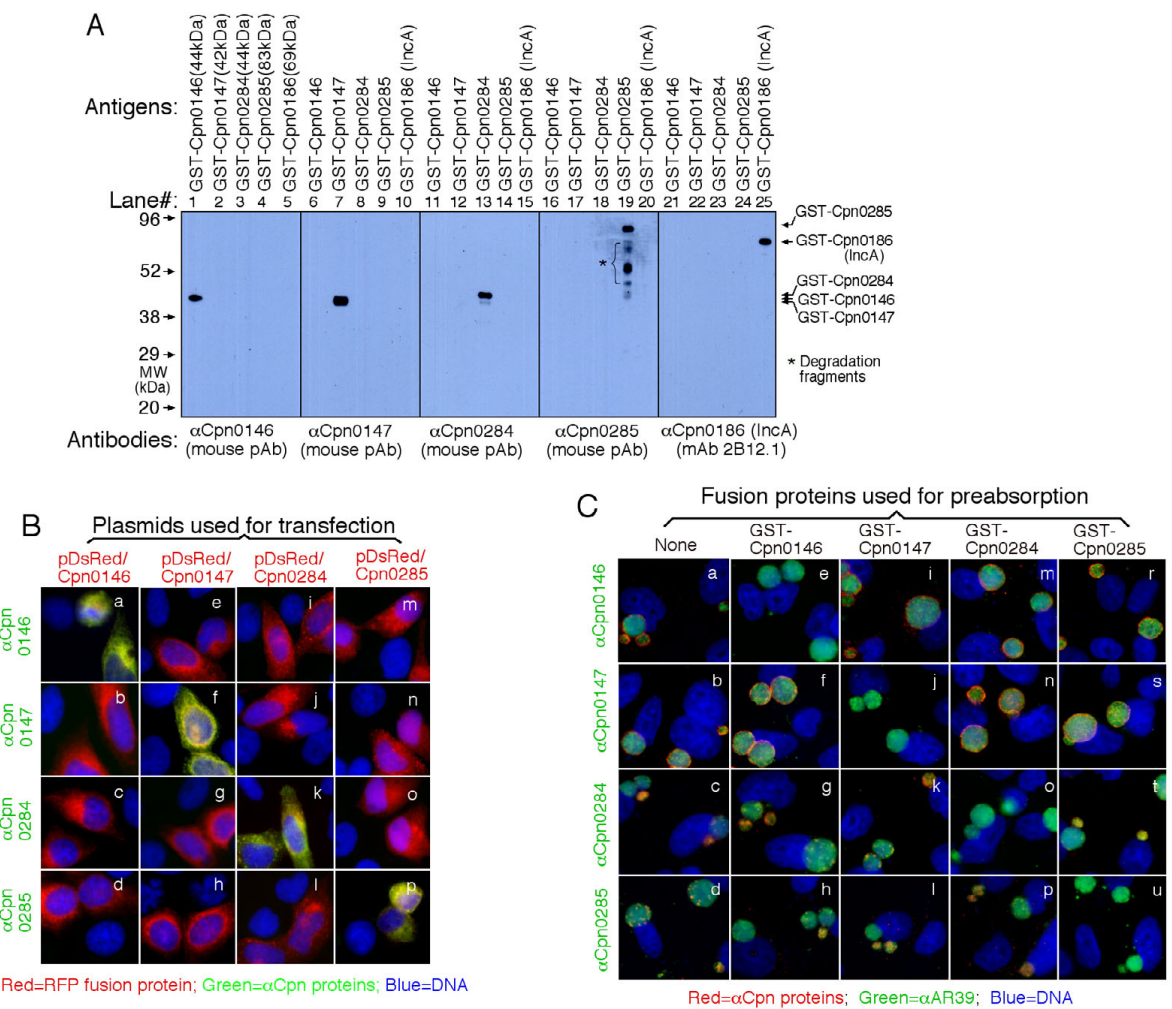
**Evaluation of the anti-Cpn fusion protein antibody binding specificities using three different methods**. (A) Various GST-Cpn fusion proteins listed on top of the figure were resolved in SDS polyacrylamide gels and the protein bands were transferred onto membranes for Western blot detection with antibodies listed at the bottom of the figure. The molecular weight (MW) was listed in kDa on the left while the corresponding fusion protein bands detected were indicated on the right of the figure. Star * denotes degradation fragments of fusion proteins. Note that the antibodies only detected the corresponding fusion proteins without cross-reacting with unrelated fusion proteins under the current experimental conditions. In separate experiments, pre-absorption of the primary antibodies with corresponding but not unrelated fusion proteins blocked the antibody binding to fusion protein bands (data not shown). (B) HeLa cells transfected with the recombinant plasmids pDsRed-C1 monomer/*Cpn0146 *(panels a-d), /*Cpn0147 *(e-h), /*Cpn0284 *(i-l) and /*Cpn0285 *(m-p) were processed for immunostaining with various antibodies listed along the left of the figure (green) plus Hoechst (Blue). The Cpn proteins were expressed as RFP fusion proteins (red). Note that the antibodies only labeled the corresponding homologous gene-transfected cells (panels a, f, k & p; yellow) without cross-reacting with the unrelated gene-transfected cells. (C) The anti-Cpn0146, 0147, 0284 and 0285 antibodies were preabsorbed with or without the GST fusion proteins listed on top of the figure followed by immunostaining as described in Fig. 1A legend. Note that antibody staining was only blocked by pre-absorption with the corresponding homologous GST fusion proteins.

Due to the fact that chlamyial antigens can be picked up by nonspecific antibodies, we further used several approaches to confirm the antibody binding specificities. First, a Western blot assay was used to measure the reactivity between the anti-fusion protein antibodies and the GST fusion proteins (Fig. 2A). The anti-Cpn0146, 0147, 0284, 0285 & 0186 (IncA) antibodies only recognized the corresponding fusion proteins without obvious cross-reaction with each other despite the common GST tag shared by all fusion proteins. This is because the titers of antibodies recognizing the chlamydial protein portions were higher than the titers of those recognizing GST tag alone in the anti-fusion protein antisera and when the anti-fusion protein antisera were diluted high enough, the anti-GST antibodies were no longer detectable. To further exclude the GST tag effect, we evaluated the reactivity between the anti-GST fusion protein antibodies and the RFP (red fluorescence protein)-*C. pneumoniae *fusion proteins expressed in transfected cells (Fig. 2B). Again, the antibodies only detected the corresponding RFP fusion proteins (panels a, f, k & p) without recognizing the unrelated proteins (e, i & m for anti-Cpn0146; b, j & n for anti-Cpn0147; c, g & o for anti-Cpn0284; d, h & I for anti-Cpn0285). Finally, the recognition of the endogenous chlamydial proteins by the anti-fusion protein antibodies was evaluated using a pre-absorption experiment (Fig. 2C). The binding to the endogenous antigens in the *C. pneumoniae*-infected cells by the four anti-fusion protein antibodies was blocked only by the corresponding homologous (e, j, o & u) but not the unrelated heterologous GST fusion proteins (i, m & r for anti-Cpn0146; f, n & s for anti-Cpn0147; g, k & t for anti-Cpn0284; h, I & p for Cpn0285). Together, the above experiments have demonstrated that the anti-fusion protein antibodies can specifically detect the corresponding endogenous antigens in the *C. pneumoniae*-infected cells.

### 3. The hypothetical proteins Cpn0146, 0147, 0284 & 0285 are unique to the *C. pneumoniae *species

**Figure 3:**
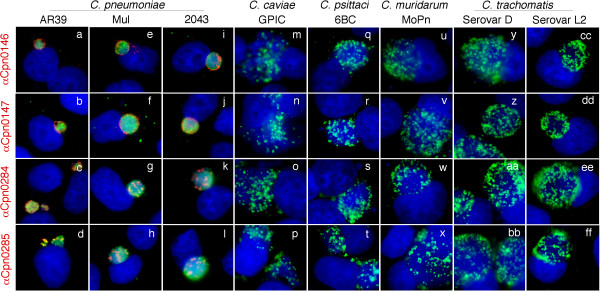
**Detection of Cpn0146, 0147, 0284 and 0285 in cells infected with different species of chlamydial organisms and identification of hydropathy regions in these 4 Cpn proteins**. HeLa cells infected with various chlamydial strains/serovars representing different chlamydial species as listed on top of the figure were processed for immunostainings with four mouse polyclonal antibodies as listed along the left side of the figure. The mouse antibody bindings were visualized with a Cy3-conjugated goat anti-mouse IgG (red) while the chlamydial inclusions were visualized by a rabbit anti-CT395 antiserum plus a Cy2-conjugated goat anti-rabbit IgG (green) and DNA by Hoechst (blue). Note that the strains Mul and 2043 are recent clinical isolates of C. pneumoniae and all four mouse antisera only detected strong signals in cells infected with the organisms of *C. pneumoniae *species but not other chlamydial species.

Cpn0146, 0147, 0284 & 0285 are listed as hypothetical proteins in the *C. penumoniae *genome sequence website [38]. Blast search has revealed no significant homologues of Cpn0146, 0147, 0284 & 0285 in any other chlamydial species [39]. We assessed whether the polyclonal antisera raised with the GST-fusion proteins can pick any signals in cells infected with other chlamydial species (Fig. 3). All four antisera detected strong signals in cells infected with three *C. pneumoniae *isolates AR39, Mul and 2043 (panels a-l) but not the *C. caviae *GPIC (m-p), *C. psittaci *6BC (q-t), *C. muridarum *MoPn (u-x) and *C. trachomatis *serovar D (y-bb) and serovar L2 (cc-ff) organisms. Since some of these antibodies seemed to preferentially recognize RBs, we also did a similar immunofluorescence assay using cells that were infected with various chlamydial organisms for only 18 hours when most intravacuolar organisms are at RB stages. Again, we found that none of these antibodies detected any significant signals in cells infected with chlamydial species rather than the *C. pneumoniae *species. These observations are consistent with the sequence homology search result that no significant homologues of these 4 *C. pneumoniae *proteins were found in any other chlamydial species [39]. Previous studies have shown that although chlamydial inclusion membrane proteins share very limited primary sequence homology, they contain a highly conserved bi-lobed hydrophobic domain [32]. Since all 4 proteins were predicted to be Inc proteins [32,33], we reanalyzed these 4 protein primary sequences with the Kyte-Doolittle hydropathy plot program [40,41]. Under this program, the hydrophobic transmembrane regions are identified by peaks with hydropathy scores greater than 1.8 when using a window size of 19 [41]. We found that IncA protein displayed two consecutive peaks with a hydropathy score above 1.8 in its N-terminal region and so did all four hypothetical proteins (data not shown) although not all were localized in the inclusion membrane (see Fig. 1).

### 4. Time course expression of Cpn0146, 0147, 0284 & 0285

**Figure 4:**
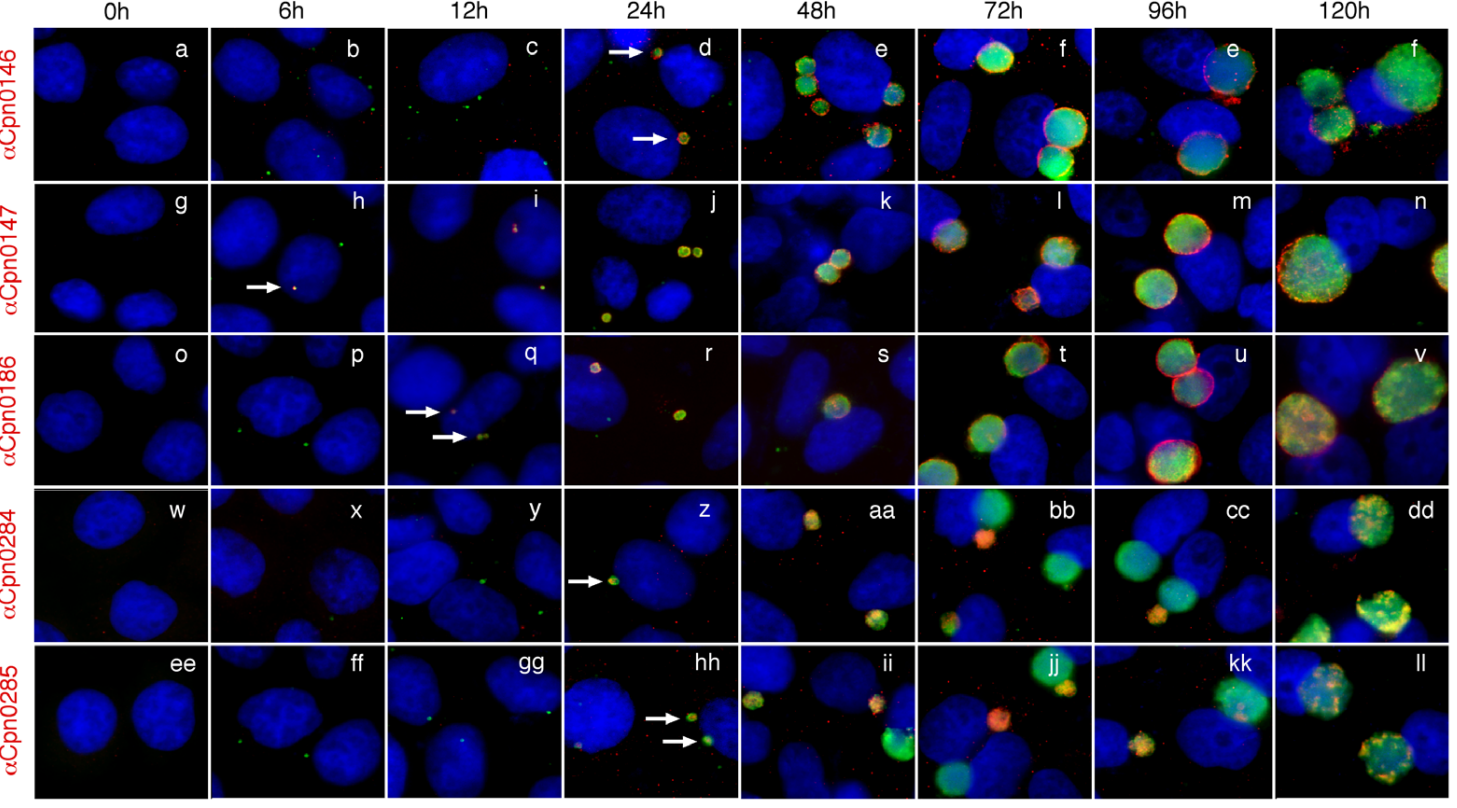
**Monitoring the expression of Cpn0146, 0147, 0284 and 0285 proteins during chlamydial infections**. HeLa cells infected with *C. pneumoniae *AR39 organisms for various periods of time as indicated on top of the figures were subjected to immunostaining with the anti-Cpn0146 (clone 3G2; panels a-f), anti-Cpn0147 (clone 2G4; panels g-n), anti-Cpn0186 (IncA; clone 2B12.1; o-v), anti-Cpn0284 (pAb; w-dd) and anti-Cpn0285 (pAb; ee-ll) antibodies as listed along the left side of the figure (red). The rabbit antibody R12AR39 was used to visualize the inclusions (green) and Hoechst dye for DNA (blue). The images were acquired using the conventional fluorescence microscope. Note that Cpn0147 protein was first detected 6 hours (panel h, white arrow), IncA 12 hours (q, white arrow), Cpn0146, 0284 & 0285 24 hours (panels z & hh respectively, white arrows) after infection with *C. pneumoniae*.

Using the specific antibodies described above, we monitored the expression patterns of both the inclusion membrane proteins (Cpn0146 & 0147) and the proteins localized inside inclusions (Cpn0284 & 0285) during chlamydial infection (Fig. 4). Cpn0147 became detectable as early as 6 hours (panel h, white arrow), IncA 12 hours (q), Cpn0146, 0284 & 0285 all at 24 hours (d, z, hh respectively) after *C. pneumoniae *AR39 infection. These observations suggest that Cpn0147 is an early protein while Cpn0146, 0284 & 0285 are late proteins. It is worth noting that the two Inc proteins Cpn0146 and 0147 remained in the inclusion membrane throughout the rest of infection cycle once they became detectable, suggesting that the Inc proteins play essential roles in chlamydial interactions with host cells throughout the infection cycles. However, the two RB proteins were only dominantly detected in small inclusions that are full of RBs and were almost absent in large inclusions that are full of EBs. This distinct distribution pattern was most obvious between 48 and 96 hours after *C. pneumoniae *infection (panels bb, cc, jj & kk). Interestingly, by 120 hours after infection, Cpn0284 and 0285 proteins reappeared in all large inclusions although in a scatter form along the inclusion periphery.

### 5. Localization of Cpn0146 & 0147 but not Cpn 0284 & 0285 in the host cell endoplasmic reticulum

**Figure 5:**
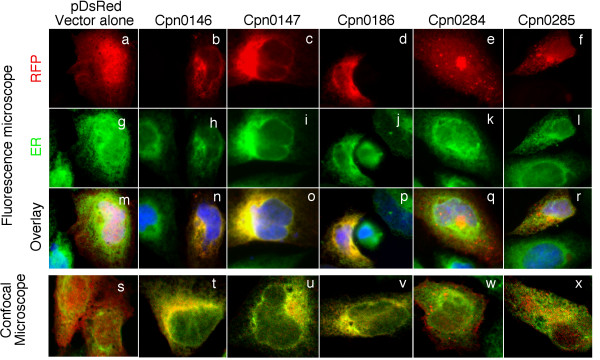
**Localization of RFP-Cpn fusion proteins in host cell endoplasmic reticulum (ER)**. HeLa cells transfected with pDsRed plasmids encoding various *C. pneumoniae *proteins as listed on top of the figure were subjected to immunofluorescence staining. The RFP or RFP fusion proteins were in red while host cell endoplasmic reticulum was labeled with a rabbit antibody against Calnexin (as an ER marker) in combination with a goat anti-rabbit IgG conjugated with Cy2 (green) and DNA labeled with Hoechst dye (blue). The slides were observed under both conventional fluorescence (panels a-r) and confocal (s-x) microscopes. Note that the microscopic observations revealed ER co-localization of Cpn0146 (panels n & t), 0147 (o & u) & 0186 (IncA; p & v) but not RFP alone (m & s), Cpn0284 (q & w) or 0285 (r & x).

**Figure 6:**
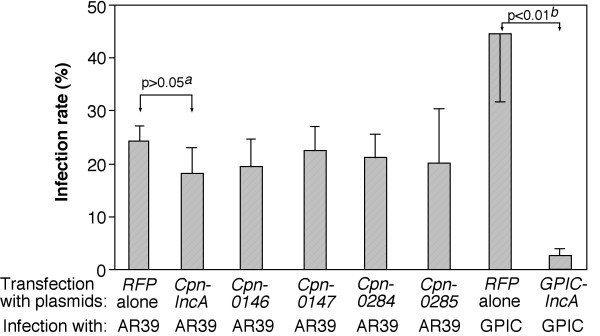
**Effects of RFP-Cpn fusion protein expression on the subsequent chlamydial infection**. HeLa cells transfected with pDsRed plasmids coding for various RFP fusion proteins as listed along the X-axis were subjected to infection with either *C. pneumoniae *AR39 or *C. caviae *GPIC organisms. 72 (for AR39) or 40 hours (for GPIC) after infection, the cultures were subjected to immunostainings for visualizing RFP fusion proteins (red), chlamydial organisms (a rabbit anti-CT395 IgG plus a Cy2-conjugaed goat anti-Rabbit IgG; green) and DNA (Hoechst, blue). The slides were viewed under a conventional fluorescence microscope. Approximately 100 red cells from 5 to 10 random views were counted in each experiment and the % of cells infected with chlamydial organisms was calculated. The results from three independent experiments were expressed as mean values plus/minus standard errors. A two-tailed Student *t *test was used to analyze the results. Note that expression of RFP-Cpn fusion proteins (including CpnIncA) failed to significantly alter the susceptibility of the transfected cells to *C. pneumoniae *AR39 organism infection while the expression of RFP-GPICIncA prevented the subsequent infection with GPIC organisms. "*a*" indicates statistically insignificant difference (P > 0.05) while "*b*" indicates a highly significant difference (P < 0.01).

It has been previously shown that IncA proteins from both *C. trachomatis *and *C. caviae *species are associated with host cell endoplasmic reticulum (ER) when expressed via transgenes [42] and the ER-localized IncA proteins can further prevent subsequent chlamydial infection [42]. We compared the cytosolic distribution patterns (Fig. 5) and the effects of the cytosolic expression on the subsequent chlamydial infection between the inclusion membrane proteins Cpn0146 & 0147 and the RB proteins Cpn0284 and 0285 (Fig. 6). When the Cpn proteins were expressed as fusion proteins with RFP as an N-terminal tag, we found that the Inc proteins Cpn0146, 0147 & 0186 (IncA) co-localized with host cell ER (Fig. 5, panels n, o & p) while the RB proteins Cpn0284 & 0285 failed to do so (panels q & r). The co-localization was confirmed with confocal microscopy (panels s-x). When the transfected HeLa cell samples were subsequently infected with *C. pneumoniae *AR39 organisms, we found that the transfected cells were similarly susceptible to the chlamydial infection regardless of whether the cells expressed RFP alone or RFP-Cpn fusion proteins (Fig. 6). For example, there was no significant difference in infection rates between cells expressing RFP alone and cells expressing RFP-IncA fusion proteins (p > 0.05) although the RFP-IncA-transfected cells displayed the lowest infection rates among the 6 transfected and *C. pneumoniae*-infected culture samples. As a positive control, when the rates of infection with *C. caviae *GPIC organisms were compared between HeLa cells expressing RFP alone and RFP GPIC-IncA fusion proteins, we found that the cells expressing GPIC-IncA were highly resistant to GPIC organism infection (Fig. 6, last 2 bars; p < 0.01), which suggests that the RFP fusion system does not interfere with the function of Inc proteins. It is clear that the cytosolically expressed Inc proteins from *C. pneumoniae *species failed to significantly alter the susceptibility of the transfected cells to the subsequent *C. pneumoniae *infection.

## Discussion

In the current study, we have provided the first experimental evidence demonstrating that the hypothetical proteins Cpn0146 & 147 are Inc proteins while Cpn0284 and 0285 are associated with RBs inside the inclusions although all 4 proteins were predicted to be in the inclusion membrane based on their N-terminal bi-lobed hydrophobic motifs [32,33]. Although the antibodies used for localizing these four proteins were raised with fusion proteins, we have convincingly demonstrated that the anti-fusion protein antibodies specifically recognized the corresponding endogenous antigens during *C. pneumoniae *infection. It is interesting that Cpn0147 was detected as early as 6 hours while IncA was only detected 12 hours and Cpn0146, 0284 & 0285 24 hours after infection. These differential protein expression patterns suggest that *Cpn0147 *and *IncA *are early genes while *Cpn0146*, *0284 *&*0285 *late genes although further monitoring transcripts is required for providing evidence to support the conclusion. Although *Cpn0146 *and *0147 *are encoded in the same gene cluster and oriented in the same direction in the *C. pneumoniae *genome, Cpn0147 is expressed much earlier than Cpn0146, suggesting that the expression of these two genes are regulated independently. Regardless of the difference in the timing of expression, both Cpn0146 and 0147 proteins remained in the inclusion membrane once detected. However, the RB proteins Cpn0284 and 0285 fluctuated along the infection cycle with strong signals in small inclusions and no or very low signals in large inclusions between 24 and 96 hours and reappeared in large inclusions by 120 hours after infection, suggesting that Cpn0284 & 0285 are RB-specific proteins although more biochemical assays are required for confirming this conclusion.

Since the identification of the first chlamydial inclusion membrane protein [21], various features have been assigned to chlamydial inclusion membrane proteins, including localization in the inclusion membrane, accessibility to host immunoproteasomal processing, secretability by various heterologous type III secretion systems, bi-lobed hydrophobic motifs, ER-like distribution and the ability to render host cell resistance to subsequent chlamydial infection when pre-expressed in host cell cytosol. Here, we systematically analyzed four predicted Inc proteins encoded by two separate gene clusters in the *C. pneumoniae *genome. We found that Cpn0146 & 0147 but not Cpn0284 & 0285 were localized in the inclusion membrane during *C. pneumoniae *infection and co-localized with ER when expressed via transgenes, which suggests that the ability of *C. pneumoniae *proteins to co-localize with ER is a more precise predictor for inclusion membrane localization than the bi-lobed hydrophobic motifs. However, more studies are required for strengthening such a correlation.

Despite the tremendous efforts that have been made in the past decade in identifying and characterizing chlamydial Inc proteins, the precise functions of the Inc proteins are largely unknown. Among the numerous Inc proteins identified in *C. trachomatis *and *C. caviae*, some Inc proteins (such as IncA) have been shown to participate in vesicle fusion [42-44] while others (such as IncG) to directly interact with host cell molecules [45-47] during chlamydial infection. Delevoye et al [42] has recently correlated the oligomerization and ER colocalization of the *C. trachomati *and *C. caviae *IncA proteins with their abilities to prevent subsequent organism infection and to disrupt the organism developmental cycle of existing infection and further mapped the functional region to the IncA C-terminal fragment that contains putative leucine zipper domains. Although *C. pneumoniae *IncA also contains the C-terminal putative leucine zipper domains [42] and has the ability to localize to ER (current study, Fig. 5), it failed to affect the subsequent *C. penumoniae *organism infection (Fig. 6), suggesting that ER localization is not sufficient for inhibiting chlamydial infection. The fact that none of the *C. pneumoniae *Inc proteins tested so far affected the subsequent *C. pneumoniae *infection suggests that Inc proteins from *C. pneumoniae *may exert their functions in different modes due to the unique growth properties of *C. pneumnoniae *organisms. We are in the process of developing novel approaches for further characterizing the *C. pneumoniae *Inc proteins.

## Methods

### 1. Cell culture and chlamydial infection

HeLa 229 cell (ATCC, Manassas, VA 20108) monolayers were infected with *C. pneumoniae *AR39, Mul or 2043 strains (kindly provided by Dr. Harlan Caldwell, RML, NIAID, NIH, Hamilton, Montana; Both Mul & 2043 strains are recent clinical isolates), *C. caviae *GPIC, *C. psittaci *6BC, *C. muridarum *(also known as MoPn) or *C. trachomatis *serovar D or L2 organisms at an MOI of 0.5 in DMEM (Invitrogen, Carlsbad, CA) with 10% fetal calf serum (FCS; Atlanta Biologicals, Lawrenceville, GA) and with or without 2 μg/ml of cycloheximide (Sigma, St. Luis, MO) for 6 to 120 hours (as indicated in individual experiments). The infected cultures grown on coverslips were processed for various immunoassays.

### 2. Prokaryotic expression of *C. penumoniae *proteins and antibody production

The open reading frames (ORFs) coding for hypothetical proteins Cpn0146, 0147, 0284 and 0285 from the *C. pneumoniae *genome [38] were cloned in full-length into pGEX vectors (Amersham Pharmacia Biotech, Inc., Piscataway, NJ) using the *C. pneumoniae *AR39 organism genomic DNA as template. We used the ORF designations described for the CWL029 genome sequence when we started the *C. pneumoniae *gene cloning/fusion protein project. In order to maintain consistence, we are still using the Cpn designations in the current study although the DNA template is from AR39 strain organisms. Please note that Cpn0146 is designated as CP0627, Cpn0147 as CP0626, Cpn0284 as CP0474 and Cpn0285 as CP0473 in the AR39 genome sequence. The amino acid sequences of these 4 proteins are identical between CWL029 and AR39. The *C. pneumoniae *proteins were expressed as fusion proteins with glutathione-s-transferase (GST) fused to the N-terminus of the chlamydial proteins as previously described [26]. Expression of the fusion proteins was induced with isopropyl-beta-D-thiogalactoside (IPTG; Invitrogen) and the fusion proteins were extracted by lysing the bacteria via sonication in a Triton-X100 lysis buffer (1%TritonX-100, 1 mM PMSF, 75 units/ml of Aprotinin, 20 μM Leupeptin and 1.6 μM Pepstatin). After a high-speed centrifugation to remove debris, the fusion protein-containing supernatants were purified using glutathione-conjugated agarose beads (Pharmacia) and the purified proteins were used to immunize mice for producing both polyclonal antisera (pAb; ref: [48] and monoclonal antibodies (mAb; ref: [49]. The fusion protein-specific antibodies were then used to localize the endogenous proteins in *C. pneumoniae*-infected cells via an indirect immunofluorescence assay [50,51].

### 3. Eukaryotic expression of *C. pneumoniae *proteins

The *C. pneumoniae *ORFs *Cpn0146*, *0147*, *0284 *&*0285 *were also cloned into the pDsRed Monomer C1 mammalian expression vector (BD Biosciences Clontech, San Jose, CA) and expressed as fusion proteins with a Red fluorescence protein (RFP) fused to the N-terminus. The recombinant plasmids were transfected into HeLa cells using the lipofectamine 2000 transfection reagent following the protocol recommended by the manufacture (Invitrogen). The RFP chlamydial fusion proteins were visualized via either the fusion tag RFP or the mouse anti-chlamydial protein antibody labeling 24 hours after transfection or as indcated in individual experiments. To assess the effects of the RFP-Cpn fusion proteins on the subsequent chlamydial infection, the transfected cells were infected with *C. pneumoniae *AR39 organisms. The infected cultures grown on coverslips were processed for visualization of the transfection and infection via an immunofluorescence assay. Cells expressing RFP were counted and % of RFP+ cells that contain chlamydial inclusions was calculated. In each experiment, ~100 RFP+ cells were counted from 5 to 10 random views and three separate experiments were carried out. The mean values were compared between the sample expressing RFP alone and samples expressing RFP-Cpn fusion proteins using a two-tailed Student *t *test. The results were expressed as means plus/minus standard errors. As a positive control, a recombinant pDsRed plasmid encoding the RFP-GPICIncA fusion protein was similarly transfected into HeLa cells followed by the *C. caviae *GPIC organism infection. The rates of GPIC infection in RFP+ cells were acquired and analyzed as described above.

### 4. Immunofluorescence assay

HeLa cells grown on coverslips were fixed with 2% paraformaldehyde (Sigma) dissolved in PBS for 30 min at room temperature, followed by permeabilization with 1% saponin (Sigma) for an additional 30 min. After washing and blocking, the cell samples were subjected to antibody and chemical staining. Hoechst (blue, Sigma) was used to visualize nuclear DNA. A rabbit anti-chlamydial organism antibody (R12AR39, raised with *C. pneumoniae *AR39 organisms, unpublished data) or anti-CT395 (raised with the CT395 fusion protein; CT395 is a GrpE-related chaperonin with >70% amino acid sequence identity among all chlamydial species; unpublished data) plus a goat anti-rabbit IgG secondary antibody conjugated with Cy2 (green; Jackson ImmunoResearch Laboratories, Inc., West Grove, PA) was used to visualize chlamydial inclusions. The mouse antibodies including both polyclonal antisera (pAbs) and monoclonal antibodies (mAbs) raised against various reference proteins and the *C. pneumoniae *GST fusion proteins plus a goat anti-mouse IgG conjugated with Cy3 (red; Jackson ImmunoResearch) were used to visualize the corresponding antigens. In some cases, the primary antibodies were pre-absorbed with either the corresponding or heterologous fusion proteins immobilized onto agarose beads (Pharmacia) prior to staining cell samples. The pre-absorption approach was carried by incubating the antibodies with bead-immobilized antigens for 1 h at room temperature (RT) or overnight at 4°C followed by pelleting the beads. The remaining supernatants were used for immunostaining. For the transfected cell samples, the RFP chlamydial fusion proteins were visualized via the fusion tag RFP (red) and by co-staining with a mouse antibody plus a Cy2 conjugate (green). For determining the subcellular location of the RFP fusion proteins, a rabbit anti-Calnexin antibody (Cat# SPA-860, Stressgen Bioreagents Corp., Ann Arbor, MI) in combination with a Cy2-conjugated goat anti-Rabbit IgG (green) was used to label the host cell endoplasmic reticulum.

The cell samples after the appropriate immuno-labeling were used for image analysis and acquisition with an Olympus AX-70 fluorescence microscope equipped with multiple filter sets (Olympus, Melville, NY) as described previously [52,53]. Briefly, the multi-color-labeled samples were exposed under a given filter set at a time and the single color images were acquired using a Hamamatsu digital camera. The single color images were then superimposed with the software SimplePCI to display multi-colors. An Olympus FluoView™ Laser Confocal Microscope (Olympus) was used to further analyze the co-stained samples at the UTHSCSA institutional core facility. All microscopic images were processed using the Adobe Photoshop program (Adobe Systems, San Jose, CA).

### 5. Western blot assay

The Western blot assay was carried out as described elsewhere [51,54]. Briefly, the chlamydial GST fusion proteins were solublized in 2% SDS sample buffer and loaded to SDS polyacrylamide gel wells. After electrophoresis, the proteins were transferred to nitrocellulose membranes and the blots were detected with primary antibodies. The primary antibody binding was probed with an HRP (horse radish peroxidase)-conjugated secondary antibody and visualized with an enhanced chemiluminescence (ECL) kit (Santa Cruz Biotechnology, Inc., Santa Cruz, CA). In some cases, the primary antibodies were also subjected to pre-absorption as described above prior to reacting with the nitrocellulose membrane.
